# Depression, anxiety, and help-seeking among Slovenian postsecondary students during the COVID-19 pandemic

**DOI:** 10.3389/fpsyg.2024.1461595

**Published:** 2024-11-13

**Authors:** Špela Selak, Maša Lebar, Gregor Žvelc, Branko Gabrovec, Andrej Šorgo, Katarina Cesar, Nuša Crnkovič

**Affiliations:** ^1^National Institute of Public Health, Ljubljana, Slovenia; ^2^Department of Psychology, Faculty of Arts, University of Ljubljana, Ljubljana, Slovenia; ^3^Faculty of Natural Science and Mathematics, University of Maribor, Maribor, Slovenia

**Keywords:** depression, anxiety, help-seeking, students, COVID-19

## Abstract

**Introduction:**

The COVID-19 pandemic has faced students with many challenges, contributing to their mental distress. This article explores the role of demographic characteristics, psychological status, year of study, and social support during the COVID-19 pandemic in explaining Slovenian university students’ mental health problems and help-seeking behavior.

**Methods:**

In February and March 2021, data were collected on 5,234 full-time Slovenian postsecondary students who were enrolled in public and concessionary independent higher education institutions. Participants completed a questionnaire, which included The Patient Health Questionnaire (PHQ-9), General Anxiety Disorder questionnaire (GAD-7), Oslo Social Support Scale (OSSS-3), and items measuring psychological status, help-seeking behavior and demographics. Data were analyzed using multiple linear regression and hypothesis testing for differences.

**Results:**

Factors, such as gender, age, psychological status and social support, contributed to experiencing anxiety and depression among Slovenian students during the COVID-19 pandemic, with year of study additionally contributing to experiencing depression. Similarly, gender, age, psychological status and social support contributed to help-seeking behavior. Higher levels of depression and anxiety were reported by female students, students with prior mental disorders, and students with lower social support levels. Female students and students with lower social support levels reported more frequent help-seeking behaviors.

**Conclusion:**

The results provide insight into the mental state of the Slovenian student population in the context of imposed public health measures at the end of the second COVID-19 wave in Slovenia. The findings can help identify vulnerable groups within the student population to whom we must be particularly attentive in times of crisis.

## 1 Introduction

Research suggests that postsecondary students in particular are exposed to a higher risk of developing mental health problems and that three-fourths of mental health issues in general occur before the age of 24 (Kessler et al., 2005). Factors, such as new responsibilities and roles, academic pressure (Yikealo et al., 2018; Racic et al., 2017), poorer financial status (Heckman et al., 2014), and uncertainty of the future (Tosevski et al., 2010), can enhance students’ feelings of emotional burden and increase psychological fluctuations (Li et al., 2021). Poor mental health in turn may negatively affect students’ attention, energy and motivation to complete academic goals (Mao et al., 2021).

During the COVID-19 pandemic, in addition to already known, new risk factors for students’ mental health have emerged. Students had to face challenges due to sudden transmission to online education (Faize and Nawaz, 2020; Virtič et al., 2021; Dolenc et al., 2021; Dolenc et al., 2022; Batra et al., 2021), and some might also struggled with Zoom fatigue, i.e., a form of anxiety caused by online human interaction (Schlesselman et al., 2020). They have also faced the loss of different job opportunities and the lack of practical experience (Mao et al., 2021). Moreover, they reported increased levels of worry regarding infection precaution measures (Šorgo et al., 2022; Velikonja et al., 2020), financial uncertainty, academic performance and delays (Dhar et al., 2020; Son et al., 2020), own and relatives’ health, disruption of daily routine and poorer social support (Khan et al., 2021). Slovenian students were mostly affected by precaution measures of limited socialization, limited use of food vouchers, cancelation of public transport, closure of faculties and student dormitories (Grosar, 2020). Many of them had to move back to their primary family environment, which may have disrupted their process of gaining independency. Some were forced into care for sick or elderly family members (Mohar, 2022). It seems that the pandemic caused more stress to students indirectly through worries about academic overload rather than directly, i.e., the actual spread of the disease and its consequences on physical health (Padrón et al., 2021; Wang et al., 2020). With all of the above, many researchers (Stylianou et al., 2020; Xiong et al., 2020) claim that the population of 21 or younger (mostly students) (Son et al., 2020; Marelli et al., 2021; Rajkumar, 2020; Solomou and Constantinidou, 2020; Zhang et al., 2022a; Zhang et al., 2022b) presents the highest risk for mental health problems during the pandemic, such as depression and anxiety. During the COVID-19 pandemic, meta-analytical evidence on mental health symptoms among students around the world show pooled prevalence of 20% (Pappa et al., 2021), 31% (Zhang et al., 2022c), 38% (Chen et al., 2021), 45% (Zhang et al., 2022a) and 50% (Zhang et al., 2022b), with lowest in Southeast Asia and highest in Spain.

One of the studies reported that during the pandemic, more than 60% of the American student sample met criteria for at least one mental health problem, most commonly depression or anxiety (Lipson et al., 2022). Moreover, an increase in the proportion of depressive and anxiety symptoms in comparison to pre-pandemic time was found (Martinez and Nguyen, 2020; Zimmermann et al., 2021). More specifically, studies reported a 10% (Fruehwirth et al., 2021), 32% (Volken et al., 2021) or even 39% increase (Li et al., 2021) in depressive symptoms. Meta-analyses on students around the world showed depression prevalence of 23% (Pappa et al., 2021), 32% (Zhang et al., 2022c), 48% (Chen et al., 2021), 54% (Zhang et al., 2022a) and 59% (Zhang et al., 2022b). Using the PHQ-9 in cross-sectional studies, the results indicated different levels of prevalence of clinically relevant (PHQ-9 ≥ 10) depressive symptoms among students, ranging from 9% [China (Tang et al., 2020)], 25% [Albania (Mechili et al., 2021)], 27% [Swiss (Volken et al., 2021)], 31% [Ukraine (Rogowska et al., 2020)], 37% [Germany (Kohls et al., 2021)], 43% [France (Essadek and Rabeyron, 2020)], 47% [United Kingdom (Van Der Feltz-Cornelis et al., 2020)], 53% [Bangladesh (Sultana et al., 2021)], 55% [Russia (Zolotareva et al., 2023)] to 66% [Spain (Padrón et al., 2021)]. The prevalence of depression among Slovenian students was 34% (Ochnik et al., 2021a).

Meta-analyses on students around the world showed anxiety prevalence of 18% (Pappa et al., 2021), 31% (Zhang et al., 2022c; Chen et al., 2021), 39% (Zhang et al., 2022b) and 43% (Zhang et al., 2022a). Studies using the GAD-7 showed different prevalence of clinically relevant (GAD-7 ≥ 10) anxiety symptoms among students, ranging from 4% [China (Cao et al., 2020)], 14% [Cyprus (Stylianou et al., 2020)], 37% [United Kingdom (Van Der Feltz-Cornelis et al., 2020)], 39% [France (Essadek and Rabeyron, 2020)] to 61% [Spain (Padrón et al., 2021)]. The prevalence of anxiety symptoms among Slovenian students ranged between 16% (Podlesek and Kavčič, 2021) and 28% (Ochnik et al., 2021a). Furthermore, Elmer and colleagues (Elmer et al., 2020) found that students who expressed more worries about the safety of their family and friends were more likely to have depressive symptoms; meanwhile, students who dealt with worries about academic goals and career pursuit experienced more anxiety during the pandemic.

Although the evidence regarding demographic risk factors for poor mental health among students, such as gender and age, is inconsistent, research results predominantly indicate that the pandemic had a higher psychological impact on women than on men (Browning et al., 2021; Ropret et al., 2023) – both in terms of prevalence of depressive (Li et al., 2021; Batra et al., 2021; Khan et al., 2021; Padrón et al., 2021; Wang et al., 2020; Fruehwirth et al., 2021; Volken et al., 2021; Essadek and Rabeyron, 2020; Sultana et al., 2021; Elmer et al., 2020; Amendola et al., 2021; Chen et al., 2020; Nomura et al., 2021; Zhou et al., 2020) and anxiety symptoms (Batra et al., 2021; Fruehwirth et al., 2021; Essadek and Rabeyron, 2020; Zolotareva et al., 2023; Elmer et al., 2020; Amendola et al., 2021), even when controlling for mental distress prior to the pandemic (Zimmermann et al., 2021). Women have also perceived a higher negative impact of the COVID-19 crisis on their mental health status and financial status, expressed more worries about completing the semester (Amendola et al., 2021) and reported greater disruption of daily activities (Zimmermann et al., 2021). Similarly, younger age posed a higher risk for the development of depressive symptoms than older (Volken et al., 2021; Van Der Feltz-Cornelis et al., 2020; Coughenour et al., 2021), while older age was a positive predictor of anxiety symptoms (Li et al., 2021; Amendola et al., 2021). Moreover, Rens et al. (2021) proposed that poorer mental health might be more related to young age, rather than student status itself. Year of study has also been found to be a contributing factor in explaining mental health problems, but the evidence is not consistent. Some authors (Ochnik et al., 2021a) found that freshmen were more vulnerable to experiencing depressive symptoms, while others (Li et al., 2021; Tang et al., 2020; Chen et al., 2020) claimed a higher prevalence of depression and anxiety among final year students. However, some studies (Tang et al., 2020) did not find age to be a risk factor for mental health problems. Furthermore, researchers have also identified pre-existing mental health issues as a risk factor for developing or worsening mental health problems during the pandemic (Zimmermann et al., 2021; Fruehwirth et al., 2021; Li et al., 2020; Ochnik et al., 2021b). Last, self-perceived low social support among postsecondary students was found to be one of the essential elements in the appearance of depressive (Khan et al., 2021; Volken et al., 2021; Kohls et al., 2021; Lai et al., 2020) and anxiety symptoms (Dhar et al., 2020; Cao et al., 2020; Amendola et al., 2021), while good social support acted as a protective factor for mental health (Ropret et al., 2023; Anyolitho et al., 2021; La Rosa and Commodari, 2023). Social support was found to be a moderator variable in the pre-COVID-19 meta-analysis (Fasihi Harandi et al., 2017) by reducing the adverse effects of mental distress through helping behaviors, emotional attention, instrumental support, sociability and feedback. Many students reported loneliness during the COVID-19 social isolation (Active Minds, 2020; Labrague et al., 2021), especially those not in a romantic relationship (Elmer et al., 2020), whereas first-year students (Vaterlaus et al., 2021) and female students (Prowse et al., 2021) reported experiencing social isolation more intensively.

When experiencing mental health problems, it is important to seek help. Previous research suggests that students are hesitant to seek professional help in times of poor mental health (Blanco et al., 2008), even though they experience more mental health problems than other populations (Kessler et al., 2005). Female students sought mental help more often, which is a general trend, as women are known to seek help more frequently than men (World Health Organization, 2017). During the pandemic, access to mental help services was limited due to public health measures, lack of knowledge about available resources, financial concerns and a lack of support services (Wang et al., 2020; Lee, 2020). Almost two-thirds of the American student sample indicated that they have not tried to reach mental services, since the pandemic made it harder to access (Martinez and Nguyen, 2020). In addition, many of them counted on their universities to provide appropriate mental help resources, but unfortunately, most of them were unavailable due to COVID-19 restrictions or the expansion of the waiting list (Salimi et al., 2023).

Taken together, the aforementioned increase in the risk factors for mental health issues for postsecondary students, limited access to mental health services and reported increase in depression and anxiety symptom prevalence during the COVID-19 pandemic, this study aimed to discover the predictors of depression, anxiety and help-seeking behavior among Slovenian postsecondary students. Additionally, differences among student groups in experiencing depression and anxiety symptoms during the COVID-19 pandemic and help-seeking behavior were explored. The following research questions were examined:

- RQ1: What role do gender, age, year of study, prior mental disorders and social support play in explaining postsecondary students’ depression, anxiety and help-seeking behavior during the COVID-19 pandemic?
- RQ2: Are there differences in experiencing depression, anxiety and help-seeking behavior among postsecondary students regarding gender, prior mental disorders and social support during the COVID-19 pandemic?

## 2 Materials and methods

### 2.1 Participants and procedure

In the present study, the targeted sample was Slovenian postsecondary students who were enrolled in a full-time postsecondary program during the 2020/21 academic year. As the questionnaire was in the Slovenian language, all participants who completed the survey were fluent in the Slovenian language. There were 7,154 respondent students, out of which part-time students and all participants with more than seven random missing responses were excluded. Examination of missing values showed a pattern of early dropout due to a long survey questionnaire (49 questions). Fewer than eight missing responses meant that most participants completed demographic information, question about seeking help, GAD-7 and PHQ-9, but omitted to answer OSSS-3 and other questions, not covered in this study. The final sample consisted of 5,234 full-time students, mostly women (72.5%). The average age of the participants was 22.85 years. The demographic characteristic of the retained participants was comparable to those of the original sample. Descriptive statistics of the sample are presented in Table 1.

**Table 1:** Descriptive statistics of the sample.

	*f*	%
Gender		
Male	1,387	26.60
Female	3,807	72.50
Year of study		
Freshmen*	1,343	25.66
Second or higher	2,538	48.49
Social support		
Poor	1,447	27.65
Moderate	2,730	52.16
Strong	1,057	20.19
Help-seeking		
Never	2,275	43.47
Rarely	878	16.77
Occasionally	1,180	22.54
Often	900	17.20
Depression		
None to minimal	1,155	22.07
Mild	1,188	22.70
Moderate	1,117	21.34
Moderately severe	924	17.65
Severe	850	16.24
Anxiety		
No to low risk	1,202	22.97
Mild	1,320	25.22
Moderate	1,078	20.60
Severe	1,634	31.22

Only first time enrolled undergraduate freshmen are counted.

All methods were carried out in accordance with all relevant guidelines and regulations, including the Declaration of Helsinki. Before commencing the study, ethical approval was obtained from the National Medical Ethics Committee of the Republic of Slovenia (NMEC), Ministry of Health (No. 0120-48/2021/3). The entire methodology was carried out in accordance with the relevant guidelines and regulations. With the help of all Slovenian universities, private faculties and student organizations, the invitation to participate in the study was disseminated to students via e-mail, the aforementioned organizations’ social media and official webpages. Before starting the questionnaire, students were informed about the various aspects of the study, including their right to voluntary participation and withdrawal without any consequences, and were notified that all gathered information will be anonymous and processed in accordance with EU and Slovenian legislation. Thereafter, the participants were asked for an informed consent. Data were collected through a self-reported online survey, which was a part of a large cross-sectional study in Slovenia and took place between February 9 and March 8, 2021.

### 2.2 Materials and instruments

The questionnaire comprised demographic variables/items, such as gender, age, year and level of study (Bologna higher education system). Furthermore, items assessing history of diagnosis of mental disorder prior to the COVID-19 pandemic and help-seeking (among mental health services, such as psychological counseling, psychotherapy and online help or talking to a friend about mental issues) since the start of the COVID-19 pandemic were added. Finally, measures for depression, anxiety and social support were included. Namely, The Patient Health Questionnaire [PHQ-9 (Kroenke et al., 2001)], a 9-item self-administered screening tool for assessing depressive symptoms during the past two weeks was used. On a 4-point Likert scale, ranging from 0 (‘Not at all’) to 3 (‘Almost every day’), a higher total score indicates a higher presence of depressive symptoms in line with the criteria of the DSM-V (American Psychiatric Association, 2013). The total score is clustered as follows: minimal (1–4), mild (5–9), moderate (10–14), moderately severe (15–19), and severe depressive symptoms (20–27) (Kroenke et al., 2001). Pre-COVID-19 research shows that the PHQ-9 has sound diagnostic properties for detecting Major Depressive Disorder for a cut-off point of 10 (Manea et al., 2012; Moriarty et al., 2015), as it reflects adequate sensitivity and specificity, although a clinical assessment by a mental health professional is required for a diagnosis. Some authors (Ransing et al., 2020) warned that the PHQ-9 might not be suitable for assessing COVID-19-related mental health issues, but other researchers (Zimmermann et al., 2021) showed a specificity and sensitivity of 0.88 for assessing clinically significant depression during the COVID-19 pandemic. The internal consistency of the PHQ-9, calculated in this study, was relatively high (*α* = 0.90).

Furthermore, a Generalized Anxiety Disorder questionnaire [GAD-7 (Spitzer et al., 2006)], a 7-item self-assessment tool for quick clinical insight of anxiety during the past two weeks, was used. It is used for screening elevated symptoms of anxiety (Kavčič and Podlesek, 2020). On a 4-point Likert scale, ranging from 0 (‘Not at all’) to 3 (‘Almost every day’), a higher total score indicates a higher presence of anxiety symptoms in line with the DSM-IV criteria (American Psychiatric Association, 1994). The total score is clustered as follows: minimal (0–4), mild (5–9), moderate (10–14), and severe anxiety symptoms (15–21) (Löwe et al., 2008). A cut-off point of 10 is recommended as an indication of generalized anxiety disorder (Spitzer et al., 2006; Plummer et al., 2016), although a clinical assessment by a mental health professional is required to diagnose and determine the type of anxiety. The internal consistency of the GAD-7, calculated in this study, was relatively high (*α* = 0.94).

Finally, an Oslo 3-item Social Support Scale [OSSS-3 (Delgard, 1996)], a 3-item self-assessment tool for addressing perceived social support was administered. On a 4- and 5-point ordinal scale, it assesses the number of surrounding people willing to help, levels of interest from one’s social network and possibility of gaining practical help. A total score indicates poor (3–8), moderate (9–11) or strong social support (12–14) (Kocalevent et al., 2018). The internal consistency of the OSSS-3, calculated in this study, was relatively low but still adequate (*α* = 0.58).

### 2.3 Statistical analysis

Data were analyzed in Excel (MS Office) and R (RStudio). After a general data review with packages car and psych, we tackled the construct’s descriptive statistics and internal consistency of the tools (OSSS-3, PHQ-9 and GAD-7). In the refined database, women were randomly coded as 1 and men as 2. Due to the research plan, only first time enrolled undergraduate freshmen versus students of every other year or level of study were compared. Therefore, only differences between freshmen students and others with this study can be addressed. To assess RQ1, multiple linear regression was carried out. To assess RQ2, various tests for assumption testing for differences between central tendencies (Wilcox test of equivalent pairs, Welch test, Wilcox robust one-way ANOVA and Kruskal–Wallis test) were performed. For that, the WRS2 package was used. In certain tests, a non-parametric approach and correction for inequality of variances were utilized. At the end of assumption testing, effect sizes were calculated and post-hoc tests were conducted.

## 3 Results

### 3.1 Descriptive statistics and analysis of individual values

Examination of mental problems at the end of the second wave of the COVID-19 pandemic in Slovenia showed that the prevalence of depression symptoms (PHQ-9 ≥ 10) was 55%, and the prevalence of anxiety symptoms (GAD-7 ≥ 10) was 52%. The mean PHQ-9 total score was 11.30 (*SD* = 7.25) and the mean GAD-7 total score was 10.40 (*SD* = 6.54). Most of the participants (52%) reported moderate social support, some reported poor social support (28%) and others reported strong social support (20%). The mean OSSS-3 total score of was 9.73 (*SD* = 2.21). Approximately two-thirds of participants (71%) indicated no prior diagnosis of any mental disorders before the COVID-19 pandemic. Those with a history of a mental disorder most frequently reported diagnosis with any type of anxiety disorder (73%) and/or depression (55%). During the COVID-19 pandemic, the majority of participants never sought help in any form.

### 3.2 Role of individual factors in explaining university students’ depression, anxiety, and help-seeking behavior during the COVID-19 pandemic (RQ1)

To assess the predictive value of factors, such as gender, age, year of study, prior mental disorders and social support, on depressive symptoms, a multiple linear regression model with five predictors was conducted (Table 2). The model explained 16.4% of the variance in the PHQ-9 total score [*F*(5, 5,226) = 205, *SE* = 6.63, *r* = 0.41, *p* < 0.01, *R*^2^_adj_ = 0.163]. All five predictors in the model were found to be statistically significant (*p* < 0.001, *p*_year of study_ < 0.01). Perceived level of social support (*β* = –0.26, *p* < 0.001) proved to be the most informative variable, with lower levels of social support indicating higher levels of depressive symptoms during the COVID-19 pandemic. The fitted regression model in standardized form was: depressive symptoms = (–0.11)*z_gender_ + (–0.09)*z_age_ + (–0.04)*z_year of study_ + (0.23)*z_prior mental disorders_ + (–0.26)*z_social support_.

**Table 2:** Multiple linear regression results with five predictors of depressive symptoms.

	*b*	SE	*t*	Lower CI	Upper CI	*β*	kspr	VIF	Tol
Intercept	26.86***	0.83	32.34	25.23	28.49	0.00			
Gender	–1.74***	0.20	–8.78	–2.13	–1.35	–0.11	0.01	1.01	0.99
Age	–0.21***	0.03	–6.63	–0.27	–0.15	–0.09	0.01	1.19	0.84
Year of study	–0.76***	0.24	–3.19	–1.23	–0.29	–0.04	0.00	1.19	0.84
Prior mental disorders	3.75***	0.21	18.24	3.35	4.16	0.23	0.05	1.02	0.98
Social support	–0.85***	0.04	–20.22	–0.93	–0.77	–0.26	0.07	1.02	0.98

b = regression coefficient; SE = standard regression coefficient error; t = Wald’s test statistical significance of regression coefficients; CI = 95% confidence interval; β = standardized regression coefficient; kspr = squared semipartial correlation; VIF = variance inflation factor; Tol = tolerance. ***p < 0.001, **p < 0.01.

To assess the predictive value of factors, such as gender, age, year of study, prior mental disorders and social support, on anxiety symptoms, a first multiple linear regression model with all five predictors was conducted. The model explained 13% of the variance in the GAD-7 total score [*F*(5, 5,226) = 156, *SE* = 6.11, *r* = 0.36, *p* < 0.01, *R*^2^_adj_ = 0.129], but year of study was not significant (*p* = 0.26) and emerged as a redundant variable. Therefore, a second model with four predictors was conducted (Table 3). The new model also explained 13% of the variance in the GAD-7 total score [(*F*(4, 5,227) = 194, *SE* = 6.11, *r* = 0.36, *p* < 0.01, *R*^2^_adj_ = 0.129]. All four predictors in the second model were statistically significant (*p* < 0.001). Prior mental disorders (*β* = 0.21, *p* < 0,001) and perceived level of social support (*β* = –0.21, *p* < 0,001) proved to be the most informative variables. Therefore, diagnosis of any mental disorder before the COVID-19 pandemic and lower levels of social support indicated higher levels of anxiety symptoms during the COVID-19 pandemic. The fitted regression model in standardized form was: anxiety symptoms = (–0.14)*z_gender_ + (–0.11)*z_age_ + (0.21)*z_prior mental disorders_ + (–0.21)*z_social support_.

**Table 3:** Multiple linear regression results with four predictors of anxiety symptoms.

	*b*	SE	*t*	Lower CI	Upper CI	*β*	kspr	VIF	Tol
Intercept	23.08***	0.76	30.49	21.60	24.57	0.00			
Gender	–2.01***	0.18	–11.05	–2.37	–1.66	–0.14	0.02	1.01	0.99
Age	–0.22***	0.03	–8.26	–0.28	–0.17	–0.11	0.01	1.00	1.00
Prior mental disorders	3.06***	0.19	16.17	2.69	3.43	0.21	0.04	1.02	0.98
Social support	–0.61***	0.04	–15.75	–0.68	–0.53	–0.21	0.04	1.02	0.98

b = regression coefficient; SE = standard regression coefficient error; t = Wald’s test statistical significance of regression coefficients; CI = 95% confidence interval; β = standardized regression coefficient; kspr = squared semipartial correlation; VIF = variance inflation factor; Tol = tolerance. ***p < 0.001.

To assess the predictive value of factors, such as gender, age, year of study, prior mental disorders and social support, on help-seeking behavior, a first multiple linear regression model with all five predictors was conducted. The model explained 5.37% of the variance in help-seeking behavior [*F*(5, 5,225) = 59.3, *SE* = 1.12, *r* = 0.23, *p* < 0.01, *R*^2^_adj_ = 0.053], but year of study was not significant (*p* = 0.12) and emerged as a redundant variable. Therefore, a second model with four predictors was conducted (Table 4). The new model explained 5.32% of the variance in help-seeking behavior [*F*(4, 5,226) = 194, *SE* = 1.12, *r* = 0.23, *p* < 0.01, *R*^2^_adj_ = 0.053]. All four predictors in the second model were statistically significant (*p* < 0.001, *p*_age_ = 0.027). Gender (*β* = –0.15, *p* < 0,001) and having a prior mental disorder (*β* = 0.14, *p* < 0,001) proved to be the most informative variables. Therefore, being a woman and prior diagnosis of a mental disorder before the COVID-19 pandemic indicated more frequent help-seeking behaviors during the COVID-19 pandemic. The fitted regression model in standardized form was: help-seeking = (–0.15)*z_gender_ + (–0.03)*z_age_ + (0.14)*z_prior mental disorders_ + (–0.08)*z_social support_.

**Table 4:** Multiple linear regression results with four predictors of help-seeking.

	*b*	SE	*t*	Lower CI	Upper CI	*β*	kspr	VIF	Tol
Intercept	3.14***	0.14	22.56	2.87	3.41	0.00			
Gender	–0.37***	0.03	–10.90	–0.43	–0.30	–0.15	0.02	1.01	0.99
Age	–0.01***	0.01	–2.21	–0.02	0.00	–0.03	0.00	1.00	1.00
Prior mental disorders	0.37***	0.04	10.63	0.30	0.44	0.14	0.02	1.02	0.98
Social support	–0.04***	0.01	–5.71	–0.05	–0.03	–0.08	0.01	1.02	0.98

b = regression coefficient; SE = standard regression coefficient error; t = Wald’s test statistical significance of regression coefficients; CI = 95% confidence interval; β = standardized regression coefficient; kspr = squared semipartial correlation; VIF = variance inflation factor; Tol = tolerance. ***p < 0.001, *p < 0.05.

An examination of the regression parameter accuracy (*b* and *SE*) in all three models reflected satisfactory accuracy. The stability of all three multiple regression models, based on comparison of sample and population variance, test characteristics and confidence intervals, was satisfactorily high.

### 3.3 Differences in depression, anxiety, and help-seeking among postsecondary students regarding gender, prior mental disorders and social support during the COVID-19 pandemic (RQ2)

With Welch’s one-way test for independent samples, the null hypothesis about the equality of the arithmetic means of depressive and anxiety symptoms in male and female students was tested. On average, female students reported higher levels of depression (*M* ± *SD* = 11.85 ± 7.29) than male students (*M* ± *SD* = 9.67 ± 6.89). The difference was statistically significant [*t*(2,587) = 10, *p* < 0.01], and Cohen’s effect size indicated a small to medium effect (*d* = 0.30). Female students also reported higher levels of anxiety (*M* ± *SD* = 11.00 ± 6.51) than male students (*M* ± *SD* = 8.58 ± 66.28). The difference was statistically significant [*t*(2,544) = 12, *p* < 0.01], and Cohen’s effect size indicated a small to medium effect (*d* = 0.38). The null hypothesis of stochastic equality of help-seeking among male and female students was tested for which we used the Wilcox one-way test for independent samples. On average, female students reported higher levels of help-seeking behaviors (*M* ± *SD* = 2.28 ± 1.17) than male students (*M* ± *SD* = 1.84 ± 1.06). The difference was statistically significant [*W*(30,000) = 10, *p* < 0.01], and Cohen’s effect size indicated a small to medium effect (*d* = 0.38).

With Welch’s one-way test for independent samples, the null hypothesis about the equality of the arithmetic means of depressive and anxiety symptoms in students with prior diagnosis of mental disorders and students without such history was tested. On average, students with any prior mental disorder reported higher levels of depression (*M* ± *SD* = 14.40 ± 7.14) than students without a history of a mental disorder (*M* ± *SD* = 10.30 ± 6.84). The difference was statistically significant [*t*(4,454) = 18, *p* < 0.01], and Cohen’s effect size indicated medium effect (*d* = 0.59). Students with any prior mental disorder also reported higher levels of anxiety (*M* ± *SD* = 12.93 ± 6.23) than students without a history of mental disorder (*M* ± *SD* = 9.64 ± 6.32). The difference was statistically significant [*t*(4,454) = 16, *p* < 0.01], and Cohen’s effect size indicated a medium effect (*d* = 0.52).

With Wilcox robust one-way ANOVA for independent samples, the null hypothesis about the equality of trimmed arithmetic means of depressive and anxiety symptoms in students in relation to perceived levels of social support was tested. The results showed that students with different levels of social support reported significantly different levels of depressive symptoms (*F* = 180.45, *p* < 0.01). The effect size, which indicates the variance of depression explained by perceived level of social support, is 14.5% and reflects a large effect. Post-hoc tests, comparing depressive symptoms by groups of students with different social support levels showed that all three groups differ significantly (*p* < 0.01). Students with poor social support reported the most depression symptoms (*M* ± *SD* = 13.99 ± 7.31), those with moderate social support reported fewer symptoms (*M* ± *SD* = 10.92 ± 6.94), and students with strong social support reported the fewest depression symptoms (*M* ± *SD* = 8.59 ± 6.72). Regarding anxiety, students with different levels of social support reported significantly different levels of anxiety symptoms (*F* = 113.86, *p* < 0.01). The effect size was 9.7%, which reflects a moderate effect. Post-hoc tests showed that all three groups differ significantly (*p* < 0.01). Students with poor social support reported the most anxiety symptoms (*M* ± *SD* = 12.29 ± 6.48), those with moderate social support reported fewer symptoms (*M* ± *SD* = 10.16 ± 6.39), and students with strong social support reported the fewest anxiety symptoms (*M* ± *SD* = 8.44 ± 6.33). To examine help-seeking behaviors in relation to social support, a robust Kruskal–Wallis test was conducted. Students with different social support levels reported significantly different help-seeking behaviors [*χ*^2^(2, 5,233) = 29.70, *p* < 0.01]. The effect size was 0.5%, which is small. Post-hoc tests showed that the groups do not all differ significantly. At 1% risk level, students with poor and strong social support and those with moderate and strong social support differ significantly, while students with poor and moderate social support do not. A closer examination of descriptive statistics shows that students with poor (*M* ± *SD* = 2.24 ± 1.18) and moderate social support (*M* ± *SD* = 2.14 ± 1.14) sought help for mental health problems more often compared to students with strong social support (*M* ± *SD* = 1.99 ± 1.13).

## 4 Discussion

Postsecondary students were globally recognized as one of the most vulnerable populations during the COVID-19 pandemic (Stylianou et al., 2020; Rajkumar, 2020; Solomou and Constantinidou, 2020; Zhang et al., 2022a; Podlesek and Kavčič, 2021; Chen et al., 2020; American Psychiatric Association, 2013; American Psychiatric Association, 1994; Löwe et al., 2008; Plummer et al., 2016; Aristovnik et al., 2020; Uzeinović, 2021). The student period itself, outside additional pressure and uncertainties surrounding the pandemic, represents a significant source of distress, due to which students are prone to bigger fluctuations in their mental state (Li et al., 2021; Storrie et al., 2010). The aim of the present study was to discover predictive factors of mental health problems and seeking help for them (RQ1) while also examining differences between gender, prior mental disorders and social support with regard to depressive and anxiety symptoms and help-seeking behavior (RQ2).

Regarding RQ1, gender, age, year of study, prior mental disorders and social support predicted 16.4% of the variance in depressive symptoms. The same predictors with the exception of year of study predicted 13% of the variance in anxiety symptoms. This indicates that being a woman, being younger, having a prior diagnosis of any mental disorder and having lower levels of social support present vulnerability to experiencing mental health issues, such as depression and anxiety. Being a freshman additionally presents vulnerability for depressive symptoms. The most informative predictor of depressive and anxiety symptoms was low social support, which is in line with prior findings (Fasihi Harandi et al., 2017), since social support reduces the adverse effects of mental distress. Regarding predictors of help-seeking behavior, only a small percentage of its variance (5.32%) was explained by gender, age, prior mental disorder and social support.

Regarding RQ2, there were significant differences in experiencing depressive and anxiety symptoms and help-seeking behaviors among different student groups. Female students reported experiencing more depressive and anxiety symptoms than male students, while female participants also reported seeking more help. This is in line with a general trend outside the crisis situations, such as pandemic, since women are more prone to experience mental distress (Pedrelli et al., 2016). Due to sex differences on the physiological level (genetic vulnerability, different hormone and cortisol levels, etc.), women can reflect different emotional and behavioral responses than men (Hankin and Abramson, 1999). Gender culture in modern society often exposes women to various discriminatory factors, such as lack of social power, underpayment and burdensome of many social roles, which may have a negative effect on their well-being and consequently mental health (Kamin et al., 2012). Women are evidently more likely to experience internalizing disorders, such as depression and anxiety, while men exhibit more externalizing disorders (Seedat et al., 2009), which were not covered in our study. Furthermore, experiencing mental health problems can be related to prescribed gender role expectations of masculinity, which manifests individualism and assertiveness, and femininity, which exhibits affection, compassion, and sensitivity (Gibson et al., 2016). By the theory of social norms, women are socialized in a manner that leads them to more likely report symptoms of psychological stress, such as tiredness, headaches, musculoskeletal problems and chronic pain (Lahelma et al., 2001). Stereotypically, it seems to be more socially and culturally acceptable for women to internalize the sick role and seek help in comparison to men who tend to overrate their overall health and conceal their problems behind the ‘tough man’ stereotype (Malnar and Hafner-Fink, 2013; Rasmussen et al., 2018). During the socialization process, women are encouraged to express and communicate their emotions, while the implicit aim of male socialization is fearlessness, toughness and courage (Chaplin and Aldao, 2013). This also applies for Slovenian students (Seršen, 2018; Zaviršek, 2022). Even when men and women share the same symptoms of depression, it is less commonly identified among men than women (Ramon et al., 2010), which also applies to Slovenian population, since Slovenian women more often than men recognize mental health (and overall health) problems and seek help in primary and secondary health care (Klanšček et al., 2009). The stigmatization of mental illnesses, especially among men, is certainly a matter of the Slovenian environment and culture (Klanšček et al., 2009) and leads to insufficiently diagnosed mental health problems in men (Kamin et al., 2012). We can draw from this that gender mechanisms could have a direct effect as female students may indeed experience higher levels of depressive and anxious moods. At the same time, there can also be an indirect connection of gender through the factors of different socialization, social isolation, education (Kamin et al., 2012), stigma and help-seeking behaviors. During the COVID-19 pandemic, female students were said to have perceived a greater deterioration of their mental state (Padrón et al., 2021; Fruehwirth et al., 2021; Volken et al., 2021; Essadek and Rabeyron, 2020; Zolotareva et al., 2023; Elmer et al., 2020; Amendola et al., 2021), a more intense experience of social isolation (Prowse et al., 2021) and also reported lower levels of psychological resilience compared to male students (Gabrovec et al., 2022). Additionally, the reduction of social support during the crises leads to a worse outcome for them than for male students (Elmer et al., 2020). From this point of view, more frequent reports of mental distress by female students may simply be a matter of greater willingness of women to report distress, as some researchers noticed even during the COVID-19 pandemic (Zimmermann et al., 2021). In addition, there is often a higher proportion of female than male students in such research, which can impact the results.

Furthermore, students with a prior diagnosis of mental disorders reported more depressive and anxiety symptoms than those without. This is in line with other studies (Zimmermann et al., 2021; Fruehwirth et al., 2021; Li et al., 2020; Ochnik et al., 2021b), since previous experiences of mental distress act as a risk factor in re-experiencing mental health problems during periods of increased stress, such as the COVID-19 pandemic. Last, students with lower levels of social support reported more depressive and anxiety symptoms in comparison to students with stronger social support. This is consistent with other research, as the absence of social support acts as a risk factor for experiencing depression (Volken et al., 2021; Kohls et al., 2021; Lai et al., 2020) and anxiety (Cao et al., 2020). On the other hand, the presence of social support acts as a protective factor against mental health problems (Amendola et al., 2021), especially in crises, such as the COVID-19 pandemic, as one perceives situation less threatening when surrounded by people whom one can turn to (Prince, 2021). Most of the participants in this study reported moderate social support during the pandemic.

Regarding help-seeking for mental distress, there were differences among students with lower levels of social support (poor and moderate levels) compared to students with strong social support. This shows that only strong social support acts as a sufficient source of help, which helps students in such extent that they do not need to seek help elsewhere. In contrast, students who do not perceive enough social support around them are more likely to seek help from professionals or online sources. In Slovenia, even before the COVID-19 pandemic, the provision of help for mental health problems was unevenly regionally distributed, understaffed, and therefore difficult to access (Maučec Zakotnik et al., 2021). During the pandemic, most Slovenian students did not seek professional help due to the perceived inaccessibility, lack of information on where and when to seek help, financial reasons, lack of willingness for accepting help and fear of the unknown (Kerč et al., 2021; Leban, 2021). Slovenian students mostly sought support from friends, acquaintances and family members, while the majority of them tried to solve emotional distress on their own (Kerč et al., 2021; Šelih, 2021), which is also shown in this study (Table 1).

Prevalence of clinical important depression symptoms (PHQ-9 ≥ 10) in this study was 55%, and the anxiety symptoms (GAD-7 ≥ 10) 52%. This is relatively high compared to other studies among Slovenian students during the COVID-19 pandemic, where 34% of students exceeded the critical threshold of experiencing depression (Ochnik et al., 2021a), and prevalence of clinical important anxiety symptoms was 28% (Ochnik et al., 2021a) and 16% (Podlesek and Kavčič, 2021). Foreign research reported even higher prevalence of experiences of depression and anxiety among students (Padrón et al., 2021; Zimmermann et al., 2021). Worldwide meta-analyses of the prevalence among students during the COVID-19 pandemic showed relatively similar prevalence of depression, with the exception of the Southeast Asia (Pappa et al., 2021), where the prevalence of anxiety was somewhat lower compared to our study (Zhang et al., 2022a; Zhang et al., 2022b; Pappa et al., 2021; Zhang et al., 2022c; Chen et al., 2021). With high prevalence rates it is necessary to consider the time specificity of the used measuring tools PHQ-9 and GAD-7 for the last two weeks. Data collection took place right during the winter exam period, which may have contributed to students’ emotional distress even outside the pandemic pressures. In addition, occasional mental upheavals during the student period are expected due to the pressures of studying, changes in habits and living environment even outside the pandemic. The high prevalence of experiencing depressive and anxiety symptoms may therefore be of a transitory nature and does not necessarily imply diagnosis of mental disorders without further individual clinical assessment of mental health experts (Malla and Gold, 2024) nor does it mean that everyone who experiences mental distress needs to seek professional help (Wang et al., 2017). Additionally, anxiety can be understood as expected, normal and adaptive response to changes during crisis situations, as it motivates people to take preventive measures (Taylor, 2022). In our study, a third of students perceived that anxiety did not affect their everyday performance in a great matter, while a third perceived a rather strong impact on their everyday life. Similar conclusions were reached by other Slovenian researchers (Podlesek and Kavčič, 2021). Nevertheless, it should be noted that high rates of emotional distress among students is present even outside the pandemic surroundings (Kessler et al., 2005; Storrie et al., 2010; Ahuvia et al., 2024). Therefore, we cannot draw conclusions about the actual (causal) impact of the pandemic without longitudinal studies outside and during the times of crisis situations.

The findings of this research can serve as a starting point for designing interventions that are specifically adapted for postsecondary students during crisis situations. On the basis of this study, actors of student mental health (e. g., universities, student organizations, counselors, public health institutions) can focus more precisely on the identified risk and protective factors within the diverse student population. Timely and appropriately implemented interventions can support students to maintain stable mental health, which is important for effective study performance.

### 4.1 Limitations

In this study, the full heterogeneity of the student population (e.g., students with special needs, part-time students, student parents, international students, and ethnic groups among students) was not considered. Therefore, it might be unfair to somewhat unite diverse individuals, as specific individual circumstances of vulnerable student groups can further contribute to worse mental health status during the COVID-19 period (Lipson et al., 2022; Fruehwirth et al., 2021; Gamage et al., 2020). Another limitation of the study could be self-election bias since the participants voluntarily chose to respond to an invitation prompted by their university. Possible differences between respondents and other students are unknown. Moreover, since this was a cross-sectional study design, we could not compare participants’ mental health status with their mental health state pre-pandemic. Due to the research plan, only first-time enrolled undergraduate freshmen were examined and compared to students of every other year or level of study. Therefore, only differences between freshmen (first-year) students and others in this study could be addressed. Additionally, we addressed help-seeking within one question, where help of a friend and an expert was considered as an equivalent answer. A distinction between these two aspects of help-seeking would provide us with much more information. Last, due to time specific of the PHQ-9 and GAD-7 instruments, the findings cannot be generalized to the entire duration of the pandemic, but must be interpreted in the context of precaution measures at the end of the second wave of the COVID-19 pandemic in Slovenia.

For further research, we strongly encourage implementation of longitudinal studies that can capture the dynamics of changes in mental health over time, especially during prolonged crises, such as the pandemics. Further research could also focus on examination of interaction effects between research variables (e.g., gender and social support) and predictive value of other factors (e. g., perceived impact of precaution measures, fear of infection, financial status, feelings of loneliness, mental health literacy, stress coping strategies, psychological resilience) that could help explain additional variance in understanding factors of depression, anxiety and help-seeking behaviors. Another important topic could also be research on barriers for not seeking help, such as institutional (absence of help within universities), sociocultural (stigma of social groups) and organizational barriers (waiting lines, financial constraints) (Dunley and Papadopoulos, 2019). In this research, we focused only on research of depression and anxiety, even though students also experienced other mental health problems, such as eating disorders (Kerč et al., 2021), post-traumatic stress disorder (Batra et al., 2021), insomnia (Zhang et al., 2022a; Zhang et al., 2022b; Chen et al., 2021), behavioral addictions (Alimoradi et al., 2022), psychosomatic illnesses (Zolotareva et al., 2023), etc. In order to better cover the gender differences in experiencing mental distress, externalizing disorders, such as substance abuse and internet addiction, which are more common in men (Seedat et al., 2009), should be included. A more detailed examination of mental distress among students in other crisis situations, such as war situations and natural disasters, could also be the subject for further research.

## 5 Conclusion

This study identified some individual factors, that made students more vulnerable to experiencing depressive and anxiety symptoms, and hesitant to seek help during the COVID-19 pandemic. Female gender, younger age, prior diagnosis of any mental disorder and lower level of social support acted as risk factors for anxiety (Table 3) and depression symptoms (Table 2), with first year of study enrolment additionally acted as a risk factor for the latter. Social support acted as the strongest predictor for explaining depressive and anxiety symptoms. As the level of social support increased, the level of experiencing depression and anxiety decreased and vice versa. The COVID-19 pandemic also contributed to the growth of differences in mental health status among different student groups. The findings are in line with other global studies carried out during the pandemic (Zimmermann et al., 2021; Fruehwirth et al., 2021; Volken et al., 2021; Essadek and Rabeyron, 2020; Elmer et al., 2020; Amendola et al., 2021; Chen et al., 2020). The prevalence of depression and anxiety were 55 and 52%, respectively, which is higher compared to other studies among Slovenian students during the COVID-19 pandemic (Zhang et al., 2022a; Zhang et al., 2022b; Pappa et al., 2021; Zhang et al., 2022c; Chen et al., 2021). The outline of Slovenian postsecondary students’ mental health during crises such as the COVID-19 pandemic can serve as a starting point for a targeted prevention and other ways of responding to the challenges students face in such periods. Poor mental health conditions of students predict lower academic success and a higher probability of dropping out (Eisenberg et al., 2009). Therefore, it is key to address students’ mental health in times such as global health crises.

## Data Availability

The raw data supporting the conclusions of this article will be made available by the authors, without undue reservation.
